# Choice of steroids for intratympanic therapy– a retrospective comparison

**DOI:** 10.1007/s00405-025-09387-9

**Published:** 2025-04-21

**Authors:** Lennart Weitgasser, Stefan Tschani, Magdalena Kogler, Maximilian Armstorfer, Wolfgang Schimetta, Sebastian Roesch

**Affiliations:** 1 Department of Otorhinolaryngology, Head and Neck Surgery, University Hospital Salzburg, Muellner Hauptstrasse 48, Salzburg, 5020 Austria; 2 Department of Clinical Pharmacy and Drug Information, Landesapotheke Salzburg, Salzburg, Austria; 3 Department of Applied Systems Research and Statistics, Johannes Kepler Universität, Linz, Austria; 4 Department of Otorhinolaryngology, Head and Neck Surgery, University Hospital Regensburg, Regensburg, Germany

**Keywords:** Sudden hearing loss, SNHL, Intratympanic therapy, Dexamethasone, Triamcinolone acetonide

## Abstract

**Purpose:**

Comparison of dexamethasone phosphate (DXA) and triamcinolone acetonide (TCA) for intratympanic therapy (IT) in patients with unilateral idiopathic sudden sensorineural hearing loss (ISSNHL) to reveal a potential superiority regarding the efficiency to improve hearing function.

**Methods:**

Retrospective, monocentric, two-armed cohort-study. Analysis of clinical and audiometric data of patients treated with IT for unilateral ISSNHL. 118 adults, who received IT with DXA (*n* = 67) or TCA (*n* = 51) were included. Main outcome parameters were hearing improvement in percent and decibel (dB) in relation to the healthy ear within a follow-up period of three months. Response to therapy was defined as an improvement of at least 10dB in affected frequencies.

**Results:**

Median improvement was 24.6% and 6.2 dB in the DXA group, 31.4% and 10.0 dB in the TCA group. For the response analysis, 7 patients dropped out. Of the remaining 111 patients, 27 (43,5%; 95%CI 30,9–56,7) in the DXA and 26 (53,0%; 95%CI 38,2–67,4) in the TCA group showed response. Analysis of the outcome parameters showed no statistically remarkable difference (*p* < 0,05) between the two cohorts. The difference of initial pure tone average between the affected and healthy ear and the time between symptom onset and first IT, was confirmed as an influencing factor to the outcome.

**Conclusion:**

For intratympanic treatment of ISSNHL dexamethasone phosphate and triamcinolone acetonide seem equivalent considering its ability to improve hearing function. A more severe hearing loss and a short duration between onset and therapy may favor the chance of hearing recovery.

**Supplementary Information:**

The online version contains supplementary material available at 10.1007/s00405-025-09387-9.

## Introduction

Among the scientific discussion on therapeutic regimes, currently used for treatment of idiopathic sudden sensorineural hearing loss (ISSNHL), selection of the active ingredient has had minor attention. However, the unique anatomical circumstances of the middle ear and the cochlea, representing the target of any therapeutic attempt, ask for best possible pharmacokinetic properties of agents used.

Intratympanic administration of synthetic steroids represents one possible mode of drug application in case of ISSNHL. Clinical impact of this route of administration has been investigated recently in a Cochrane Review by Plontke et al. [1]. Besides a generally low or very low certainty of evidence for effectiveness of this intervention, authors were able to identify at least low-certainty evidence for a higher proportion of patients with improvement of hearing loss and a small effect on a change of hearing thresholds, when used as sole secondary therapy (without combined systemic steroids) compared to placebo or no treatment. With no further pharmaceutical alternatives at hand, in case of insufficient primary therapy, intratympanic steroids provide at least a therapeutic option. The active ingredient– the type of synthetic steroid used for intratympanic therapy (IT)– varies among different sites.

The route of administration into the inner ear via the middle ear, using the round window for passive penetration, asks for special pharmacokinetic properties of the active ingredient. Two main steps after application are relevant for best possible efficacy in the inner ear. First, entrance to the perilymph by overcoming the round window and second, continuance of the agent within the inner ear [2]. The agents most commonly used for intratympanic application have been dexamethasone phosphate (DXA) solution and triamcinolone acetonide (TCA) suspension, both synthetic steroids of liquid aggregate state. Both agents have been investigated for their pharmacodynamic properties in the context of intratympanic application [3, 4]. TCA shows better characteristics concerning penetration into the perilymph, due to lipophilic properties, however, elimination from the inner ear is much quicker for TCA compared to DXA.

In accordance with a recommendation by the American Academy of Otolaryngology– Head and Neck Surgery, intratympanic administration of steroids has been provided to patients as off-label salvage sole therapy in case of unsatisfactory primary systemic therapy with prednisolone [5]. Prior to 2019, DXA has been used exclusively at our site, fabricated by the in-house pharmacy. Due to procedural issues and reported pharmacokinetic properties concerning influx into the inner ear a switch towards TCA, commercially available and ready to use, was executed.

The aim of this retrospective study was to provide data from a comparison between DXA and TCA as active ingredients, used separately during different time periods in an otherwise comparable clinical setting, concerning indication and execution.

## Materials and methods

### Study design

This is a retrospective, monocentric, two-armed cohort-study. Prior to execution a study protocol was established, allowing for traceable data acquisition and analysis. The protocol can be found as supplemental material. Patients who suffered from ISSNHL and received IT with either DXA or TCA were analysed and compared, regarding improvement of hearing function. Up until April 2019, DXA was used exclusively for IT for ISSNHL. DXA was produced at the local pharmaceutical institute on request. After an in-house evaluation, intratympanic therapy with commercially available TCA (pre-filled syringe) was introduced, due to an easier accessibility and handling. From April to August 2019 there was a transitional period wherein both substances were used, and after August 2019 TCA has been used solely. For the DXA group all cases of ISSNHL who received IT between January 2016 and August 2019 were analysed. For the TCA group the period between April 2019 and June 2022 was analysed. The period was chosen to assure constant and comparable relations between the two groups considering the treatment process and setting in the clinic, and to receive an adequate number of cases. Possible candidates were searched via the hospital information system with the integrated search engine scanning all medical reports for the key word “intratympanic” (German: intratympanal). Patients who met the selection criteria were enrolled in the study. Demographic data and contributing factors were collected based on the medical history. In general, all audiometric data of our department is stored in a separate electronic data processing system (Innoforce, ENTstatistics©). Pure tone audiograms only were examined, no speech discrimination scores. Storing and processing of all demographic and audiometric data for the analysis was done with Microsoft Excel©.

### Inclusion and exclusion criteria

Patients included for analysis had to meet the following criteria. Only adult patients aged 18 years or older were included. All patients presented with unilateral ISSNHL, matching the 2014 German criteria of *Arbeitsgemeinschaft der wissenschaftlich medizinischen Fachgesellschaften (AWMF)– Leitlinie Hörsturz* [6], and received three intratympanic applications of either DXA or TCA for therapy. Onset of symptoms had happened no longer than three months prior to the first intratympanic application. Audiometric data at hand for each patient included at least one pure tone audiogram before therapy (PRE), one after therapy (POS) and one follow-up audiogram (FOL) within three consecutive months after therapy.

Patients aged younger than 18 years and cases with reliable causes of hearing loss such as toxic damage due to infection (bacterial or viral, e.g. zoster oticus) or medication, hydropic inner ear disease (Menière´s disease according to AAO-HNS guidelines [7]), hereditary hearing loss, vestibular schwannoma, malformations of the inner ear or cochlear hemorrhage diagnosed by radiological imaging, acute trauma or previous surgery of the ear were excluded from analysis.

### Intratympanic therapy

IT comprised a daily application of either DXA or TCA for three consecutive days. Recommended reporting of precise compositions of agents used [8] are as follows: DXA was produced by our pharmaceutical department and consisted of a 0.6 ml solution containing 4 mg dexamethasone-21-dihydrogenphosphate and 0.1 mg sodium hyaluronate. TCA is a purchasable manufactured product in a concentration of 40 mg per ml, available in an ampulla or a pre-filled syringe. Local anaesthesia at that time was done in two different ways, depending on the attending physician. Mostly, a solution of 10% lidocaine or 10% lidocaine-base was applied to the tympanic membrane and the auditory canal around 20 min prior to IT, providing full coverage (e.g. by pump spray, directly poured or using a small piece of cotton). In some cases (e.g. when the solution did not have enough anaesthetic effect, or tympanic perforation was present), anaesthesia was achieved by an injection of 1% lidocaine + epinephrine 1:200.000 (manufactured product) into the roof of the auditory canal, targeting the region of the petrosquamous fissure.

DXA or TCA was administered into the middle ear through the anterior-superior quadrant of the tympanic membrane with a 20-gauge, 60 mm needle. Intratympanic injections were generally performed in a lying position with the head elevated 30 degrees. In this position the anterior-superior quadrant is the most cranial region of the tympanic membrane and therefore, injections of liquids can achieve a maximal filling of the middle ear cavity. Furthermore, the risk of injury of middle ear structures and unwanted drainage of fluid after the injection is very low. After injection, the patients remained lying with their head turned to the contralateral side for 30 min and were advised not to speak or swallow deliberately, to reduce efflux through the Eustachian tube.

### Diagnostic tympanotomy

In case of functional deafness (hearing loss of > 80 dB in all frequencies) despite three performed intratympanic injections, we provided diagnostic surgical tympanostomy due to the potential existence of a perilymphatic fistula, additionally to IT therapy. Preoperative MRI scans of the cerebellopontine angle and CT-scan of the temporal bone were routinely performed at our clinics in case of profound hearing loss. Surgery included exploration of the middle ear via an endaural approach and coverage of vulnerable regions for perilymph leakage (round and oval window niche, fissula ante fenestram region) with autologous connective tissue; this procedure was also done in case of missing obvious clinical signs or macroscopic leakage, respectively. Tympanotomy was performed in case of persistent deafness (POS audiogram) after IT therapy. As with any surgical procedure, written informed consent of the patient was obtained. In the DXA group 23 (34.33%) patients and in the TCA group 13 (25.49%) patients underwent surgery. Follow-up findings after a tympanostomy were considered as implausible values and were converted into missing values for later statistical analysis.

### Assessment and Documentation of hearing function

Storing and processing of demographic and audiometric data was done with Microsoft Excel©. A number of approximately 50 to 100 subjects in each of the two cohorts was assumed to be suitable for preliminary statements about any possible outcome differences between the two cohorts. We followed the definitions of Chandrasekhar et al. [5], who defined an improvement post-therapy if the level of the affected frequency increased by at least 10 dB. For analysis of hearing improvement, the methods of Mühlmeier et al. [9] were applied. The difference between the affected ear and the healthy ear before and after therapy, single frequencies and four-frequency pure tone average (PTA) of 500, 1000, 2000 and 4000 Hz, was determined and compared with each other. Improvement was calculated for dB and percentage as follows: e.g. a PTA of 50 dB of the affected ear compared to 20 dB of the healthy ear results in a difference of 30 dB. After therapy PTA of the affected ear is 35 dB, which defines an improvement of 15 dB. The percentage hearing improvement is calculated by dividing 15 dB by 30 dB, which results in 0.5, meaning a relative gain of hearing of 50%. The maximum dB value for measurement of the hearing threshold in our clinic is set at 120 dB. This value was also used if a patient did not react at all during the audiometry, interpreted as complete deafness.

Following parameters were defined as primary and secondary endpoints:

Primary endpoints were defined as: (a) percentage of hearing improvement based on Mühlmeier et al. [9], including the contralateral ear as a reference, for comparison between PRE vs. POS and PRE vs. FOL and (b) improvement of pure tone average, comparing PRE vs. POS and PRE vs. FOL.

Secondary endpoints included (a) improvement per frequency for PRE vs. POS and PRE vs. FOL; (b) hearing level for all frequencies after therapy (POS); (c) hearing level for all frequencies during follow-up (FOL). Contributing factors identified and incorporated during analysis were: history of previous systemic corticosteroid therapy, time period (days) between PRE and POS audiogram; time period (days) between PRE and FOL audiogram; time period (days) between POS and FOL audiogram; time period (days) between onset of symptoms and first IT; age of participant (years); sex of participants; differences in hearing level compared to healthy ear for all frequencies (dB); difference in PTA (dB) compared to healthy ear and hearing levels of the healthy ear for all frequencies.

### Statistical methods

For the comparison of DXA and TCA not only the total collective but also a bias-reduced subset was used. This was obtained by means of propensity score matching with the following variables: age; sex; difference PTA PRE vs. healthy ear; time between onset and first IT; time between audiogram PRE and POS; time between audiogram PRE and FOL; previous systemic therapy. To ensure good matches, a maximum allowable difference between two patients of 0.8 was defined.

All data of continuous variables were checked for normal distribution (test of normality: Kolmogorov-Smirnov with Lilliefors significance correction, type I error = 10%). Continuous variables with normally distributed data were compared by the t-test for independent samples. For comparisons of continuous variables without normally distributed data, the exact Mann-Whitney U test was used. Dichotomous variables were compared by Fisher’s exact test; two-sided 95% confidence intervals (95%CI) were calculated according to Clopper-Pearson).

The influence of DXA vs. TCA, age, sex, the difference of PTA PRE vs. healthy ear, the time between onset and first IT, the time between audiogram PRE and POS or PRE and FOL, and the systemic therapy, on the difference of PTA PRE vs. POS and PRE vs. FOL was investigated by multiple linear regression analyses.

The type I error was not adjusted for multiple testing. Therefore, the results of inferential statistics are descriptive only. Statistical analyses were performed using the open-source R statistical software package, version 4.2.3 (The R Foundation for Statistical Computing, Vienna, Austria).

## Results

**Fig. 1:**
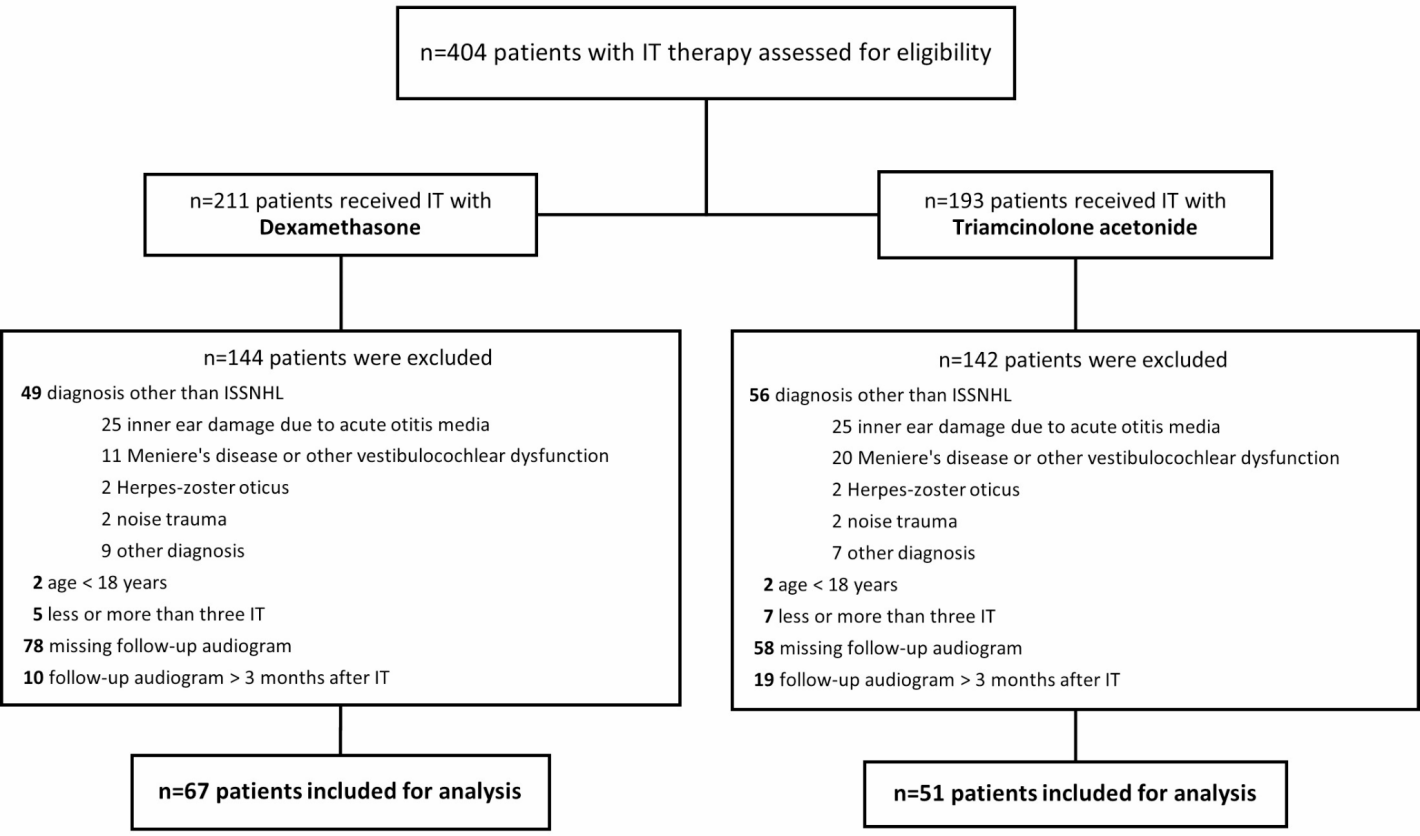
Study enrolment process; IT = intratympanic, ISSNHL = idiopathic sudden sensorineural hearing loss

In total, 404 potential patients were identified during the screening process within the defined periods, resulting in an inclusion number of 118 patients (65 male; 53 female) (Fig. 1).

67 (31 male; 36 female) patients received DXA and 51 (34 male; 17 female) TCA. Mean age was 56.9 years (max. 84.3; min 25.7), 58.2 (max. 84.3; min 26.4) in the DXA group and 55.1 (max. 84.0; min. 25.7) in the TCA group. 107 (90.6%) patients were treated with systemic corticosteroids before, 65 (97.0%) in the DXA group and 42 (82.3%) in the TCA group. Systemic therapy consists of an intravenous dose of 250 mg prednisolone, applied daily on three consecutive days. Partially, systemic therapy was done by external ENT physicians, who administered the patient to our hospital in case of persistent hearing loss. Two patients in the DXA group and 9 patients in the TCA group did not receive systemic corticosteroids before IT due to comorbidities like uncontrolled diabetes or hypertension.

The average time from onset of symptoms to the audiogram before the first IT was 9.8 days in the DXA group and 8.1 days in the TCA group. Time until the first IT was 9.9 days in the DXA group and 8.5 days in the TCA group. The mean time between the audiogram before (PRE) and after (POS) the completed therapy was 3.5 days in the DXA group and 4.3 days in the TCA group. Between the audiogram before therapy and at follow-up (FOL) we measured a mean of 32.8 days in the DXA group and 29.8 days in the TCA group.

**Fig. 2:**
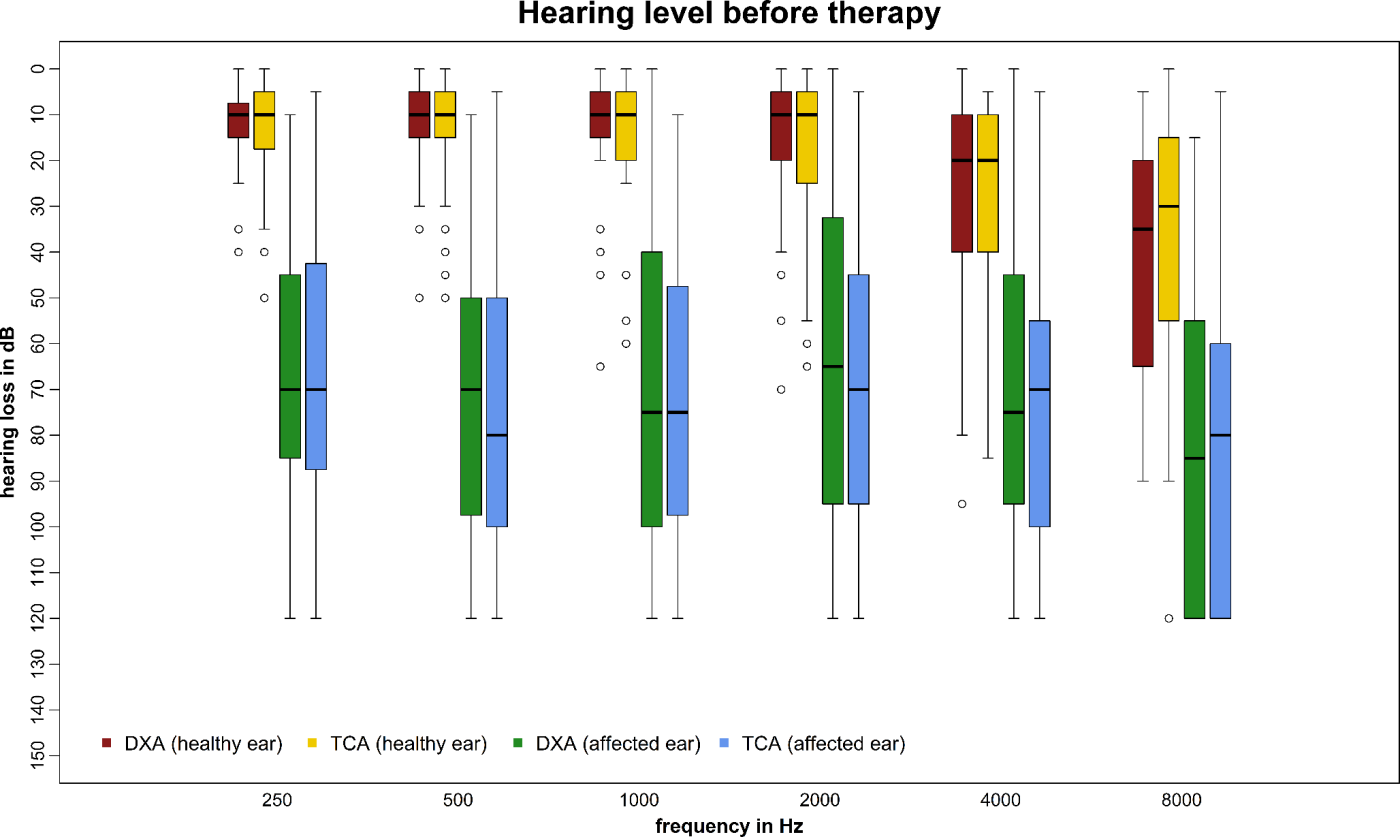
Initial hearing level of single frequencies of affected and healthy ears before intratympanic therapy; visualized by boxplots (median, interquartile range, minimum, maximum and outliers); dB = decibel; Hz = hertz; DXA = dexamethasone phosphate; TCA = triamcinolone acetonide

The median PTA of the affected ear prior to IT was 70.0 dB (max. 120.0; min 3.7) in the DXA group and 73.7 dB (max. 120; min. 12.5) in the TCA group. Figure 2 demonstrates dB values per frequency of the affected and the healthy ear. Healthy ears had a median PTA of 13.7 (max. 66.2; min. 0.0) in the DXA group and 13.7 (max. 62.5; min 1.2) in the TCA group.

**Fig. 3:**
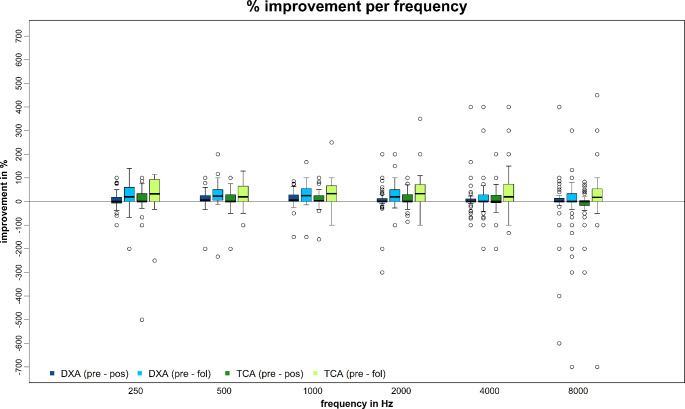
Percentage improvement of hearing function of single frequencies after therapy (PRE– POS) and at follow-up (PRE– FOL); visualized by boxplots (median, interquartile range, minimum, maximum and outliers); dB = decibel; Hz = hertz; DXA = dexamethasone phosphate; TCA = triamcinolone acetonide

**Fig. 4:**
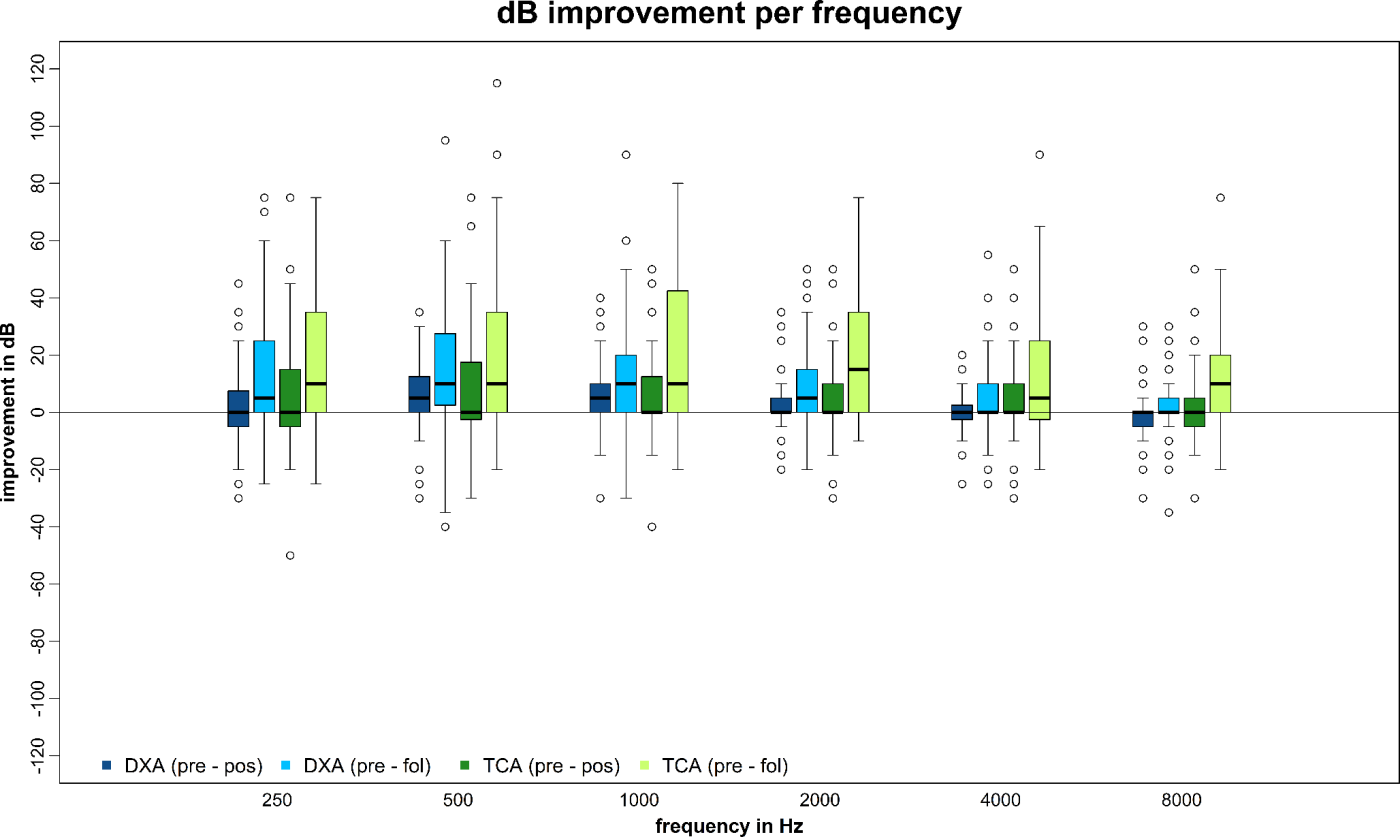
Improvement of hearing function in decibel of single frequencies after therapy (PRE– POS) and at follow-up (PRE– FOL); visualized by boxplots (median, interquartile range, minimum, maximum and outliers); dB = decibel; Hz = hertz; DXA = dexamethasone phosphate; TCA = triamcinolone acetonide

The median percentage improvement of PTA after therapy (PRE vs. POS) in the DXA cohort was 2.7% (max. 89.4; min. -400.0) and in the TCA group 1.8% (max. 130.0 min. -144.4). Improvement at follow-up (PRE vs. FOL) was 24.6% (max. 100.0; min. -433.3) in the DXA group, and 31.4% (max. 300.0; min. -66.6) in the TCA group. PTA in dB after therapy improved by 1.2 dB (max. 28.7; min. -25.0) in the DXA cohort and 1.2 dB (max. 47.5; min. -32.5) in the TCA cohort. At follow-up it was 6.2 dB (max. 71.2; min. -21.2) in the DXA and 10.0 dB (max. 86.2; min. -7.5) in the TCA group. Individual dB and percentage values for each frequency are shown in Figs. 3 and 4.

For the response analysis 7 patients (5 DXA, 2 TCA) had to be excluded because the difference between PTA of the affected ear before therapy and the healthy ear was less than 10 dB (responder = improvement ≥ 10 dB). After therapy 13 patients of 62 (20.9%; 95%CI 11.6–33.1) in the DXA cohort, 15 patients of 49 (30.6%; 95%CI 18.2–45.4) in the TCA cohort, and overall, 28 of the 111 patients (25.2%; 95%CI 17.4–34.3) showed response. At follow-up the number of responders in the DXA cohort was 27 (43.5%; 95%CI 30.9–56.7), in the TCA cohort 26 (53.0%; 95%CI 38.2–67.4) and overall, 53 (47.7%; 95%CI 38.1–57.4).

Propensity score matching resulted in a study population of 88 with each 44 patients in the DXA and TCA group, respectively. For the response analysis, 6 patients dropped out (4 DXA, 2 TCA) due to the reasons mentioned above in the overall collective. The median PTA of the affected ear before IT was 69.3 dB (max. 120.0; min 12.5) in the DXA and 72.5 dB (max. 120; min. 12.5) in the TCA group. Healthy ears had a median PTA of 13,7 (max. 66,2; min. 0.0) in the DXA and 13.7 (max. 62.5; min 2.5) in the TCA group. The median percentage improvement of PTA after therapy was 6.5% (max. 89.4; min. -400.0) in the DXA and 9.4% (max. 130.0 min. -144.4) in the TCA group. Improvement at follow-up was 25.3% (max. 100.0; min. -433.3) in the DXA and 23.6% (max. 300.0; min. -66.6) in the TCA group. PTA in dB after therapy improved by 2.5 dB (max. 28.7; min. -25.0) in the DXA cohort and 1.8 dB (max. 47.5; min. -32.5) in the TCA cohort. At follow-up improvement of PTA was 6.8 dB (max. 45.0; min. -21.2) in the DXA and 7.5 dB (max. 86.2; min. -7.5) in the TCA group.

In the response analysis 9 patients of 40 (22.5%; 95%CI 10.8–38.4) in the DXA, 14 patients of 42 (33.3%; 95%CI 18.6–39.0) in the TCA group, and overall, 23 of the 82 patients (28.0%; 95%CI 17.4–34.3) showed improvement. At follow-up 19 responders (47.5%; 95%CI 31.5–63.8) were counted in the DXA, and 21 (50.0%; 95%CI 34.1–65.8) in the TCA group. Overall, 40 (48.7%; 95%CI 37.5–60.0) patients showed response.

Analysis of the outcome parameters for the overall and propensity score matched collective showed no statistically remarkable difference (*p* < 0.05) between the DXA and the TCA cohort. Only for 8000 Hz a noticeable difference of hearing improvement after therapy and at follow-up was detected in the TCA group compared to the DXA group.

In the multivariate analyses (multiple linear regressions) the difference between PTA of the affected ear before therapy and the healthy ear, as well as the interval of symptom onset to the first IT, could be confirmed as an influencing factor to the final hearing improvement at follow.

up. A higher difference of PTA between PTA before therapy and follow-up was generally observed in cases with high differences of PTA between both ears (affected and unaffected ear) before therapy and cases of lower time-delay between onset and prior application of IT therapy, independent from the agent administered.

In both cohorts no complications (e.g. persisting perforation of tympanic membrane, infection etc.) after IT were reported during the follow-up period. Possible complications were evaluated by reviewing patients’ medical records and audiometric data like follow-up impedance audiometry.

## Discussion

The mode of treatment for ISSNHL with synthetically produced corticosteroids varies greatly among countries, sites, and individual physicians. The choice of drug and dosing, method of application, and duration of a therapy seem almost arbitrary. In consideration of the general low level of evidence for the treatment of ISSNHL with these substances, this matter is not surprising.

Based on the persistent uncertainty on the effect of corticosteroids on hearing restoration, variable studies and systematic reviews have been conducted. Those deal with the variable regimens, doses, types of application and different agents for primary therapy [1, 10–21]. To our knowledge, this is the first work, comparing the effectiveness between DXA and TCA applied intratympanically for salvage treatment of ISSNHL at a single centre, providing best possible consistency in indication, performance, follow-up and data storage.

DXA or TCA are commonly used for IT administration to treat ISSNHL [1, 21]. There are different reasons for the choice of one specific agent, like availability, costs (manufactured vs. self-made), or simply historical reasons. The fact that we experienced a switch from DXA to TCA in our clinic and that all corresponding audiometric data was available and stored, led to the possibility to evaluate a potential superiority of one of the two corticosteroids.

One challenge, during the enrolment process of candidates for this study was the fact that in most patients, audiometric follow-up after intratympanic therapy was done externally in an ENT physician´s office. In these cases, no follow-up audiogram was available. A prior attempt to ​​request hearing tests from office-based colleagues, was quickly discarded due to the enormous administrative effort needed (e.g. individual data protection declaration for every external request, postal traffic) and the inconsistent quality of the hearing tests (various audiometers used in the offices, different software, sound booth set up etc.). Therefore, many patients had to be excluded during the screening process since follow-up hearing tests were performed elsewhere. The estimated sample size of 50 to 100 per group has been achieved.

Analysis of results did not show any statistically remarkable difference between both substances regarding both, primary and secondary endpoints. Neither the improvement of PTA nor single frequencies did reveal a superiority of DXA or TCA, respectively. Only at 8000 Hz, TCA performed better (*p* < 0.05). Certainly, this hardly noticeable difference ​could have been generated by chance. Still, this observation may be of interest, regarding stated pharmacokinetic properties of TCA, with a better permeability into the perilymphatic space of the cochlea, and frequency representation within the cochlea for high frequencies in a closer proximity to the round window. However, this potential pharmacokinetic advantage of TCA may be negligible since TCA is eliminated from the perilymph rather quickly [4].

Considering the response to therapy (at least 10 dB improvement), a higher number of responders in both pre vs. pos and pre vs. fol analysis was observed in the TCA cohort (DXA 20.9% vs. TCA 30.6% and DXA 43.5% vs. TCA 53.0%). These findings were confirmed in the propensity score matched comparison.

According to the multivariate regression analyses a more severe hearing loss and a short duration between symptom onset and therapy seem to favour the chance of hearing recovery, and consequently, success of the therapy. Though, a relevant influence of the respective type of therapy (DXA or TCA) on the outcome could not be confirmed in the models used.

The potential factor of spontaneous recovery, independent from the type of steroid administered, has to be taken into account as well, during discussion of a potential effect of steroids [22]. However, this factor may influence both groups similarly and can therefore be neglected in this specific study design.

There have been published two studies recently on IT for ISSNHL comparing DXA and TCA. In contrast to this study, Emami et al. and Meybodian et al. investigated hearing improvement after IT with DXA and TCA as a primary therapy for ISSNHL. Both studies were randomized clinical trials, which is a clear advantage compared to this retrospective investigation. However, presentation of data was very different, e.g. Emami et al. categorized a response to therapy in “complete, partial and no response”, no detailed numerical data was provided [19, 20]. 

Unlike in the works of Emami et al. and Meybodian et al., in this study no speech discrimination scores were obtained [19, 20]. The reason for this was that on the one hand, speech discrimination tests were not routinely performed in every patient and on the other hand, different language skills and partly language barriers existed in the study population, which made a reliable evaluation difficult. Advantages of this study are the higher number of subjects, the performed multivariate regression analyses, and the propensity score matching resulting in an enhanced homogeneity of the cohorts. Nevertheless, further prospective studies, ideally with an additional control group and audiological follow-up including acoustic and electrophysiological examinations, are needed.

### Personal perspective on IT handling

To ensure a maximum chance of penetration of the applied corticosteroid into the cochlea, effective injection with a sufficient amount of agent to cover the middle ear cavity completely (at least a fluid level above the round window) is crucial. From a personal perspective, the white colour and viscosity of TCA, in contrast to the clear and liquid appearance of DXA, facilitate traceability of a sufficient intratympanic application, and further on, allows instant recognition of a potential descent of the fluid level if the patient swallows during application.

### Potential influence of additional tympanostomy

Within the propensity score-matched collective, diagnostic tympanostomy was distributed almost exactly equally between the two cohorts. If it had any influence on the outcome parameters, it would not play a role in the cohort comparison. If tympanostomy would have been included as an additional covariable in the regression analyses, perhaps an influence could be shown, but the main question of the study - whether the choice of corticosteroid has an influence or not– would still be answered negatively.

### Pharmacological aspects

From a chemical-pharmaceutical point of view, lipid solubility, size, polarity and water solubility are essential for the effectiveness of a substance. Lipophilic, small and apolar molecules pass through the membrane more easily than hydrophilic, large and polar molecules. To be effective in the inner ear, the substances should have the following properties: sufficient solubility, good penetration into the perilymph and conversion into a slowly eliminating form.

DXA is larger and more polar than dexamethasone. DXA is more soluble but does not cross the lipid membrane as easily. In the inner ear, phosphatases split off the polar phosphate group, resulting in the active base form dexamethasone. Dexamethasone is less polar and smaller. It therefore passes more easily through the lipid membrane although is quickly eliminated from the perilymph. Dexamethasone has poor solubility and can therefore only be used in low concentrations.

TCA is also less polar and less soluble in water but has a high potency. According to previous examinations, TCA may have a two to six times higher potency than dexamethasone at the glucocorticoid receptor. It easily crosses the lipid membrane but is also quickly eliminated. TCA is metabolised to triamcinolone in the perilymph. Triamcinolone is less potent but more hydrophilic, more polar and therefore more soluble in water. It is also less permeable and is therefore eliminated more slowly from the perilymph [4,23–25].

For intratympanic use, DXA has been used as an aseptic magistral preparation. A clear advantage of magistral preparation is a certain degree of variability in the formulation (e.g. ingredients and dosage), although this involves a great deal of manufacturing effort due to hygiene regulations. The production of magistral medicines in pharmacies is generally based on the pharmacopoeia, the Medicines Act, and the Pharmacy Operating Regulations. In some pharmacies, production is also carried out according to GMP guidelines. TCA is already available as a ready-to-use suspension. The advantages here are the simpler application and the immediate availability of the finished drug. A disadvantage of the use of triamcinolone is the preservative benzyl alcohol that can cause local side effects such as dryness and irritation of the mucosa [26,27].

## Conclusion

This retrospective study did not confirm a considerable difference in the efficacy of restoring hearing function between dexamethasone phosphate and triamcinolone acetonide for salvage intratympanic therapy of idiopathic sudden sensorineural hearing loss. There has been observed a slight superiority of triamcinolone concerning the frequency of 8 kHz during follow-up. The number of responders was higher in the group of triamcinolone acetonide recipients. In both cohorts a higher degree of hearing loss and a shorter duration between symptom onset and IT seem to have a positive effect on the final hearing improvement. Data provided may be used for solid sample size estimation for future randomised controlled trials.

## Electronic supplementary material

Below is the link to the electronic supplementary material.

Supplementary Material 1

## Data Availability

Data supporting the findings and conclusions of this study are available on request from the corresponding author. The shared data consists of deidentified participants’ demographic and audiometric data and statistical analysis. A detailed study protocol is attached as supplementary material.
