# Supplementary material for: Choice of steroids for intratympanic therapy– a retrospective comparison
Source: Eur Arch Otorhinolaryngol. 2025 Apr 21;282(9):4579–88. doi: 10.1007/s00405-025-09387-9 (PMC12423152; doi:10.1007/s00405-025-09387-9)
Supplement: Supplementary file 1 — Supplementary Material 1 [file 405_2025_9387_MOESM1_ESM.docx]

**Study protocol**

*Translated to English from original version in German*

**Comparison of intratympanic therapy for acute hearing loss -dexamethasone phosphate vs. triamcinolone acetonide - a retrospective study**

**Introduction**

Intratympanic therapy (IT) with corticoids is a common method for the treatment of idiopathic sudden sensorineural hearing loss (ISSNHL) and is used in most cases as a secondary therapy or "salvage" therapy in case of persistent hearing loss after previous systemic corticoid therapy. The therapy is mainly carried out with the pharmaceutical agent dexamethasone phosphate (DXA) or triamcinolone acetonide (TCA). To date, there is no clear recommendation as to whether one of the two substances is preferable for use. In literature there are numerous studies, which investigate hearing improvement after treatment with one of the two active substances (1, 2). To our knowledge, there is no study to date that compares the effectiveness of intratympanic salvage therapy with DXA and TCA in patients with ISSNHL.

**Study objective**

By investigating hearing improvement after IT with DXA or TCA in patients with unilateral ISSNHL, initial results shall be obtained to proof whether one of the two active substances has a clinically relevant advantage regarding its efficacy.

**Study design**

Retrospective, monocentric, two-armed cohort-study.

2 cohorts: intratympanic therapy with dexamethasone or triamcinolone acetonide

**Bias discussion**

The use of DXA and TCA during different time periods with only a minimal duration of parallel use causes advantages and disadvantages in terms of bias potential. On the one hand, there were de facto no differential indications between DXA and TCA, which counteracts a selection bias. On the other hand, time effects (e.g. influences of changes in personnel and equipment in the study center; influences of behavioral changes in the study population due to changes in information procurement, corona pandemic, etc.) cannot be excluded.

Known (potential) influencing factors are considered for the propensity score matching and multiple regression analyses.

**Methods:**

**Selection of study participants**

Since August 2019, only TCA has been used in our clinic for IT in patients with ISSNHL. Until April 2019, only DXA was used (transition phase with parallel use of both substrates from April to August 2019).

For the DXA cohort, the search was conducted in the period from January 2016 to August 2019, for the TCA cohort from April 2019 to June 2022. The period of the DXA treatment was chosen so that the conditions were constant and comparable to the period for the TCA treatment (no deliberate modifications in patient care and environment), and that a sufficient number of cases can be expected.

In the relevant periods, all patients who meet the selection criteria (meeting all inclusion criteria, not meeting all exclusion criteria) will be included in the study.

The cohort sizes will be approx. 50-100 patients, based on estimated availability.

Potential study candidates are identified via the hospital information system (HIS) using the program's internal search function (German keyword "intratympanal"). Each patient can only be included in the study once.

Necessary demographic data and co-variables are collected on the basis of the medical history (see below). All audiometric data are stored in a separate database (ENT-Statistics) and can be analyzed and exported (Excel). Only pure-tone audiograms are examined, no speech recognition tests (see study parameters).

**Inclusion criteria**

- Unilateral idiopathic sudden sensorineural hearing loss, proven audiometrically, meeting the 2014 German criteria of *Arbeitsgemeinschaft der wissenschaftlich medizinischen Fachgesellschaften (AWMF) – Leitlinie Hörsturz (akuter idiopathischer sensorineuraler Hörverlust)* (3)
- Three intratympanic applications of either dexamethasone phosphate or triamcinolone acetonide
- At least one pure tone audiogram before (pre) and after (pos) therapy, as well as a follow-up audiogram (fol) within the next three months.
- Onset of symptoms not more than three months ago from the date of the first intratympanic application
- Participants must be at least 18 years of age

### **Exclusion criteria**

- Participants under 18 years of age
- Verified cause of hearing loss:
  - Toxic inner ear damage due to infection or medication
  - Hydropic inner ear disease (Menière) – diagnosis based on the 2020 AAO-HNS Guidelines (7)
  - Genetic hearing loss
  - Vestibular schwannoma or other retro-cochlear disease
  - Traumatic hearing loss
  - Acoustic trauma
  - Previous surgery of the middle or inner ear

**Study parameters**

**Primary outcome parameters**

- - Percentage hearing improvement based on Mühlmeier et al. (4) including the opposite ear as a reference. Improvement was defined as at least 10 dB increase in affected frequencies; pre vs. pos and pre vs. fol (%)
  - Improvement of pure tone average; pre vs. pos and pre vs. fol (dB, %)

**Secondary outcome parameters**

- Improvement per frequency; pre vs. pos and pre vs. fol (%, dB)
- Hearing level after IT, all frequencies; pos (dB)
- Hearing level at follow-up, all frequencies; fol (dB)

**Contributing factors / covariates**

- Previous systemic corticosteroid therapy (yes / no)
- Time between 1. and 2. audiogram (days)
- Time between 1. and 3. audiogram (days)
- Time between 2. and 3. audiogram (days)
- Time between onset of symptoms and first IT (days)
- Age (years)
- Sex (male / female)
- Hearing ability - difference to healthy ear (all frequencies; dB, %)
- Hearing ability - difference to healthy ear (PTA; dB, %)
- Hearing ability of healthy ear (all frequencies; dB)

**Biometric experiment design and analysis**

**Case number justification:**

The expected number of approx. 50-100 cases in each of the two cohorts should be suitable for preliminary conclusions on any outcome differences between the two cohorts.

**Collective analysis:**

As no qualitative differentiation of inclusions is planned, these are all classified as valid cases (total collective = per-protocol collective) and evaluated as part of a per-protocol analysis.

The following cohorts are defined:

- Cohort D = treatment with dexamethasone phosphate
- Cohort T = treatment with triamcinolone acetonide

To minimize bias, propensity score matching is also performed for the two cohorts (Cohort D_PS_ ; Cohort T_PS_ ):

- Cohort D_PS_ vs. cohort T_PS_ = 1:1
- Matching variables
  - Previous systemic cortisone therapy [yes / no]
  - Time difference 1st audio and 2nd audio [days]
  - Time difference 1st audio and 3rd audio [days]
  - Time difference 2nd audio and 3rd audio [days]
  - Time difference between onset of symptoms and start of IT therapy [days]
  - Age [years]
  - Gender [m / f]
  - Hearing difference to the opposite ear (average) [%]

**Implausible values and missing values:**

- Implausible values:

Implausible values are identified by the study management and converted into missing values. Implausible values include follow-up findings after a diagnostic tympanostomy.

- Missing Values:

Missing values are not replaced, with the following exception:

- Missing follow-up findings after performing a tympanostomy (between end of treatment and follow-up) are replaced using Last Observation Carried Forward (LOCF).

**Presentation of results:**

All data collected from the medical records for the study are tabulated with the number of observed and missing values.

- Nominal-scale data is presented in tables with absolute and relative frequencies.
- Ranking data is presented in tables with absolute and relative frequencies and/or using median, quartiles, minimum and maximum.
- For quantitatively measured data, the following characteristic values of their distribution are presented:
- Minimum
- Median
- Quartiles
- Maximum
- Mean value
- Standard deviation

If required, graphics (box plots and bar charts) can be created.

**Statistical analysis:**

Cohort comparisons (D vs. T and D_PS_ vs. T_PS_ ):

- Metric parameters: If the hypothesis of a normal distribution cannot be rejected (Kolmogorov-Smirnov test with Lilliefors significance, α = 10%), the respective parameter is statistically analysed using the two-sample t-test for independent samples. Otherwise, the Mann-Whitney U-test is used.
- Ordinal parameters: Statistical comparisons are made using the Mann-Whitney U-test.
- Nominal parameters: Nominal-scale data are analysed using Fisher's exact test or the chi-square test.

Regressions:

Multiple linear regression analyses are performed with all main outcome parameters (dependent variables) with the following independent variables:

With all approaches:

- - Previous systemic cortisone therapy [yes / no]
  - Time difference between onset of symptoms and start of IT therapy [days]
  - Age [years]
  - Gender [m / f]
  - Hearing ability difference to the opposite ear (average) [%]
  - Cohort ([D vs. T]

For "pre-post" approaches:

- - Time difference 1st audio and 2nd audio [days]

For "pre-fol" approaches:

- - Time difference 1st audio and 3rd audio [days]

For "post-fol" approaches:

- - Time difference 2nd audio and 3rd audio [days]
  - (only for improvement average:) Improvement average (PTA) (pre-post) [dB, %]
  - (only for percentage improvement based on Mühlmeier et al. including the opposite ear as a reference:) percentage improvement based on Mühlmeier et al. including the opposite ear as a reference (pre-post) [%]

Estimation of the true effect size:

Two-sided 95% confidence intervals are calculated for selected parameters (depending on the nature of the data: parametric, non-parametric or Clopper-Pearson).

Post-hoc analyses:

Should constellations arise after completion of the planned evaluations that make one or more post-hoc analyses (e.g. subgroup comparisons, further regression analyses, etc.) appear useful, such analyses are possible.

Alpha error level:

There is no adjustment of the alpha error level for multiple testing, so all statistical statements are purely descriptive.

**Declaration of possible conflicts of interest**

The persons conducting this study confirm that there are no conflicts of interest of a personal, financial or commercial nature.

**Financing**

This study does not receive any third-party funding or financing. For the biometric planning and evaluation, expenses are reimbursed via research funds from the ENT clinic.

**Ethical aspects**

This is a retrospective study with evaluation of primarily numerical data. The data of the patients concerned are processed and evaluated exclusively in pseudonymized form. Prior to pseudonymization, only persons who are authorized to do so as part of their professional routine know the patients by name (i.e. no data entry, creation, maintenance and storage of a specific study participant identification list by other personnel).

No conclusions can be drawn about personal data or even the identity of individual patients on the basis of the results presented.

**Publication of the results**

The study results should be published in an internationally recognized scientific journal in the field of ear, nose and throat medicine or audiology and neurotology.

**References**

1. Salt AN, Plontke SK. Pharmacokinetic principles in the inner ear: Influence of drug properties on intratympanic applications. Hear Res. 2018;368:28-40.

2. Salt AN, Hartsock JJ, Hou J, Piu F. Comparison of the Pharmacokinetic Properties of Triamcinolone and Dexamethasone for Local Therapy of the Inner Ear. Front Cell Neurosci. 2019;13:347.

3. S1 Guideline: sudden hearing loss (acute idiopathic sensorineural hearing loss) [Internet]. Association of the Scientific Medical Societies in Germany (AWMF) - Standing Committee Guidelines. 2014. Available from: [https:](https://www.awmf.org/leitlinien/detail/ll/017-010.html)//www.awmf.org/leitlinien/detail/ll/017-010.html.

4. Mühlmeier G, Maier S, Maier M, Maier H. [Intratympanic injection therapy for therapy refractory acute hearing loss: A safe option for secondary treatment]. ENT. 2015;63(10):698-700, 2-6.
